# Generating and Reversing Chronic Wounds in Diabetic Mice by Manipulating Wound Redox Parameters

**DOI:** 10.1155/2014/562625

**Published:** 2014-12-23

**Authors:** Sandeep Dhall, Danh C. Do, Monika Garcia, Jane Kim, Seyed H. Mirebrahim, Julia Lyubovitsky, Stefano Lonardi, Eugene A. Nothnagel, Neal Schiller, Manuela Martins-Green

**Affiliations:** ^1^Department of Cell Biology and Neuroscience, University of California, Riverside, 900 University Avenue, Riverside, CA 92521, USA; ^2^Bioengineering Interdepartmental Graduate Program, University of California, Riverside, 900 University Avenue, Riverside, CA 92521, USA; ^3^Division of Biomedical Sciences, University of California, Riverside, 900 University Avenue, Riverside, CA 92521, USA; ^4^Department of Botany and Plant Sciences, University of California, Riverside, 900 University Avenue, Riverside, CA 92521, USA; ^5^Department of Computer Science and Engineering, University of California, Riverside, Riverside, CA 92521, USA

## Abstract

By 2025, more than 500 M people worldwide will suffer from diabetes; 125 M will develop foot ulcer(s) and 20 M will undergo an amputation, creating a major health problem. Understanding how these wounds become chronic will provide insights to reverse chronicity. We hypothesized that oxidative stress (OS) in wounds is a critical component for generation of chronicity. We used the db/db mouse model of impaired healing and inhibited, at time of injury, two major antioxidant enzymes, catalase and glutathione peroxidase, creating high OS in the wounds. This was necessary and sufficient to trigger wounds to become chronic. The wounds initially contained a polymicrobial community that with time selected for specific biofilm-forming bacteria. To reverse chronicity we treated the wounds with the antioxidants *α*-tocopherol and N-acetylcysteine and found that OS was highly reduced, biofilms had increased sensitivity to antibiotics, and granulation tissue was formed with proper collagen deposition and remodeling. We show for the first time generation of chronic wounds in which biofilm develops spontaneously, illustrating importance of early and continued redox imbalance coupled with the presence of biofilm in development of wound chronicity. This model will help decipher additional mechanisms and potentially better diagnosis of chronicity and treatment of human chronic wounds.

## 1. Introduction

Wound healing is a dynamic process involving many factors and cell types including soluble mediators, blood cells, fibroblasts, endothelial cells, and extracellular matrix. Normal wound healing is divided into several sequential phases that overlap in space and time: homeostasis, inflammation, granulation tissue formation, and tissue remodeling. Chronic wounds develop as a result of defective regulation of one or more of the complex molecular and biological events involved in proper healing [1–3]. Diabetic foot ulcers and other similar chronic wounds impact approximately 6.5 M people and cost approximately $25B/year in the US alone [4]. The critical need for a cure of chronic wounds is underlined by the continuous increase in type II diabetes which accounts for 90–95% of all diabetes [5]. Challenges in understanding these problematic events result from varying disease etiologies, existing comorbidities and, importantly, lack of animal models that mimic the characteristics of chronic wounds in humans. The importance of developing such models has been recognized by the National Institute of General Medical Sciences call for proposals in this field. Furthermore, difficulties with human tissue collection complicate chronic wound research; for the most part, a clinician sees the patient when the wound is already at an advanced stage of chronicity and critical evidence of causality is already lost.

Although the series of events leading to development of chronic wounds remains unclear, toxic concentrations of reactive oxygen species (ROS) and presence of biofilm-producing bacterial colonies are considered as key players [6–10]. The db/db mouse model of type II diabetes has impaired healing [11] with decreased levels of reduced glutathione (GSH) [12], which results in reduction of glutathione peroxidase (GPx) activity and an increase in ROS. The latter promote leukocyte adhesion, thereby increasing immigration of inflammatory cells into the wound tissue [13]. It has also been suggested that ROS impair keratinocyte migration* in vitro*, potentially inhibiting reepithelialization [14]. High levels of ROS also lead to DNA damage, gene dysregulation, cell death, and a hostile proteolytic environment [15]. Moreover, chronic wounds in humans are rich in microbiota, including anaerobic bacteria not revealed by culture that can form biofilms and cause excessive bioburden in these wounds [8, 9].* Staphylococcus aureus*,* Enterococcus faecalis*,* Pseudomonas aeruginosa*, coagulase-negative staphylococci, and* Proteus* species are among the most commonly cultured species in human chronic wounds [16].

We hypothesize that manipulating specific redox parameters immediately after wounding will lead to development of chronic wounds in db/db mice and that restoring the antioxidant status will reverse chronicity and lead to proper healing. Here we show that inhibition of the activity of GPx and catalase, two antioxidant enzymes, immediately after wounding generates chronic wounds containing spontaneously formed antibiotic-resistant polymicrobic bacterial biofilms. Moreover, chronicity can be reversed by treatment with the antioxidants N-acetyl cysteine (NAC) and *α*-tocopherol (*α*-toc). This novel model for chronic wounds provides insight into the mechanisms involved in chronic wound development and may contribute to development of new therapeutics.

## 2. Methods

### 2.1. Animals

C57BL/6 and db/db mice (Jackson Laboratories; Bar Harbor, ME) were housed in the UCR vivarium and used following protocols approved by the Institutional Animal Care and Use Committee (IACUC).

### 2.2. Dermal Excision Wound Model, Preparation of Tissue for Extracts and for Histology

The procedures used were previously described by us [10]. Briefly, mice were anesthetized with a single intraperitoneal injection of ketamine (80 mg/kg body weight)/xylazine (16 mg/kg body weight). Full thickness 7 mm punch wounds (excision of the skin and the underlying panniculus carnosus) were made on the back of the mice. The animals were euthanized using carbon-dioxide at various time points and the wound tissue was collected for histology and protein analysis using a 10 mm punch (wound bed + surrounding tissue). For protein analysis, zirconium oxide beads weighing approximately the same as the wound tissue were added to tissue in safe lock tubes, followed by addition of 10 mL of RIPA buffer per mg of tissue. The tissues were bullet blended for homogenization. The extracts were then centrifuged at 14000 rpm for 15 minutes at 4°C. The supernatants were used fresh or aliquots prepared and stored at −80°C for later use. The samples were normalized to protein levels.

### 2.3. Superoxide Dismutase Activity Assay

Total tissue superoxide dismutase (SOD) activity was measured by using a commercially available kit (Cayman Chemical, Catalog number 706002, Ann Arbor, USA) that measures all three types of SOD (Cu/Zn-, Mn-, and EC-SOD). One unit of SOD is defined as the amount of enzyme needed to cause 50% dismutation of the superoxide radical. Briefly, xanthine oxidase and hypoxanthine generate superoxide radicals that are dismutated by SOD and in the process tetrazolium salt are converted to a formazan dye that is read at 450 nm. The SOD activity of the samples was calculated from the linear regression of a standard curve that was determined using the SOD activity of bovine erythrocytes at various concentrations run under the same conditions. The SOD activity was expressed as U/mL of tissue extract.

### 2.4. Hydrogen Peroxide Activity Assay

Tissue hydrogen peroxide (H_2_O_2_) levels were measured by using a commercially available kit (Cell Technology Inc., Catalog number FLOH 100-3, Mountain View, USA) that utilizes a nonfluorescent detection reagent. The assay is based on the peroxidase-catalyzed oxidation by H_2_O_2_ of the nonfluorescent substrate 10-acetyl-3,7-dihydroxyphenoxazine to a fluorescent resorufin. 50 *µ*L of tissue extracts collected at different time points after wounding and normalized to protein concentration was mixed with 50 *µ*L of the reaction cocktail in an opaque 96-well assay plate. Fluorescent intensities were measured at 530 nm (excitation)/590 nm (emission) using a Victor 2 (fluorescence and absorbance) microplate reader. The amounts of H_2_O_2_ in the supernatants were derived from a seven-point standard curve generated with known concentrations of H_2_O_2_.

### 2.5. Catalase Activity Assay

Tissue catalase activity was measured by using a commercially available kit (Cayman Chemical, Catalog number 707002, Ann Arbor, USA). The enzyme assay for catalase is based on the peroxidatic function of catalase with methanol to produce formaldehyde in the presence of an optimal concentration of H_2_O_2_. The formaldehyde produced was measured spectrophotometrically, with 4-amino-3-hydrazino-5-mercapto-1,2,4-triazole (purpald) as the chromogen, at 540 nm in a 96-well place. The catalase activity was expressed as nmol/min/mL of tissue extract.

### 2.6. Glutathione Peroxidase Activity Assay

A commercially available kit (Cayman Chemical, Catalog number 703102, Ann Arbor, USA) was used to measure tissue glutathione peroxidase (GPx) activity. The activity was measured indirectly by a coupled reaction with glutathione reductase (GR). GPx reduces H_2_O_2_ to H_2_O and in the process oxidized glutathione (GSSG) is produced that in turn is recycled to its reduced state by GR and NADPH. Oxidation of NADPH to NADP+ is accompanied by a decrease in absorbance at 340 nm. Under conditions in which GPx activity is rate limiting, the rate of decrease in the absorbance measured at 340 nm, in a 96-well plate at 1 min interval for a total of 5 min using a Victor 2 microplate reader, is directly proportional to the GPx activity of the sample. GPx activity was expressed as nmol/min/mL of tissue extract.

### 2.7. Chronic Wound Model

To generate chronic wounds in db/db mice we performed full thickness 7 mm diameter excision wounds on the dorsum of 6-7-month-old mice. Twenty minutes prior to wounding, mice were treated once intraperitoneally (IP) with 3-amino-1,2,4-triazole (ATZ) (Aldrich Chemistry; St. Louis, MO) at 1 g/kg body weight, an inhibitor for catalase. Immediately after wounding, they were treated* once* topically with the inhibitor for GPx, mercaptosuccinic acid (MSA), (Sigma Lifesciences; St. Louis, MO) at 150 mg/kg body weight. Immediately after wounding, the wounds were covered with tegaderm (3 M; St. Paul, MN) to prevent contamination and were kept covered for the duration of the experiments. In these mice it is easy to fully remove the hair from the back and hair grows very slowly; hence we had no problems keeping the tegaderm in place. The tegaderm was removed periodically to take pictures of the wound and then immediately replaced. The wounds were fully chronic 20 days after wounding and remained open sometimes for more than 3 months, depending on the experiment.* Control db/db mice* were treated exactly the same way but instead of inhibitors of the antioxidant enzymes they were treated with the vehicle (PBS). To reverse chronicity, at 20 days, the antioxidant NAC (Aldrich Chemistry (St. Louis, MO)) was topically applied to the wound at 200 mg/kg and the tegaderm replaced. Simultaneously, the mice were injected intraperitoneally with *α*-toc, Sigma Lifesciences (St. Louis, MO) at 50 mg/kg. This treatment continued with NAC applied to the wound topically every day using an insulin syringe to deposit the solution under the tegaderm and over the wound and with *α*-toc IP every other day for 20 days (40 days after wounding). At this point, the antioxidant treatment was stopped and the wounds went on to heal around 30 days after initiation of treatment with antioxidants (50 days after wounding). For the antioxidant controls, the mice were treated exactly the same but with vehicle rather than antioxidants. In some experiments, tissues were collected at various time points for detailed histological/histochemical, biochemical, and cellular/molecular evaluation, and in some cases the tissues were analyzed for type and level of bacterial infection/biofilm production. Chronic wounds were successfully created in over 100 animals.

### 2.8. Bacteria Isolation and Characterization

To obtain the wound microbiome samples we used sterile cotton Q-tips to swab the wound bed, including the surface of the wound, but yet minimizing disruption of the wound microenvironment to allow for longitudinal studies of the microbiome. The content of each swab was suspended in 1.0% w/v protease peptone and 20.0% v/v glycerol solution. Wounded tissue for bacteria analysis was obtained using sterile scissors and suspended in 1.0% w/v protease peptone and 20.0% v/v glycerol solution. Tissues were homogenized in the presence of zirconium oxide beads using a bullet blender at 4°C. Bacteria were cultured for 16–18 h at 37°C on tryptic soy agar plates (BD Difco, Sparks, MD), containing 5.0% v/v defibrinated sheep blood (Colorado Serum Company, Denver, CO), and 0.08% w/v Congo red dye (Aldrich Chemistry, St. Louis, MO). Colonies were differentiated and isolated based on size, hemolytic pattern, and Congo red uptake. Resulting cultures were examined using Gram stain and visualized with optical microscopy. Gram-negative rods were characterized using the API20E identification kit (Biomerieux, Durham, NC) and oxidase test (Fluka Analytical, St. Louis, MO). When required, the* Pseudomonas* Isolation Agar culture test, 42°C growth test in tryptic soy broth (TSB) (BD Difco, Sparks, MD), and motility test were used. Gram positive cocci cultures were differentiated based on catalase activity and coagulation activity (Fluka Analytical, St. Louis, MO), 6.5% w/v NaCl tolerance test, and hemolytic activity. Biofilm production was quantified using methods described previously [17] with minor modifications. Briefly, 3–5 *μ*L of the wound swabbed sample was seeded in 100 *μ*L of TSB and grown in humidified incubator at 37°C in a 96-well polystyrene flat bottomed tissue culture plate under static condition. Bacterial content was removed by inverting and gently flicking the plate. The plate was washed three times by slowly submerging the plate and gently flicking the inverted plate to remove the water. The wells were dried by tapping onto absorbent paper and then air dried at 65°C for 30 minutes. The plate was cooled and stained with Hucker crystal violet [18] for 5 minutes. Excessive stain was removed by rinsing the plate with water and then air dried overnight. The optical density at 570 nm was then taken using the SpectraMax M2e microplate reader (Molecular Device, Sunnyvale, CA). Samples that give an OD of or above 0.125 were considered biofilm positive whereas OD below 0.125 was considered biofilm negative.

### 2.9. Viable Bacteria Cells Count

Wound swab samples were resuspended in sterile Luria broth (LB) to yield a 1 : 4 v/v ratio of sample-to-TSB solution. Bacterial colonies were visually counted on trypticase soy agar plates containing 5% sheep red blood cells incubated at 37°C overnight in a humidified incubator.

### 2.10. Community Minimal Inhibitory Concentrations Assay

Wound swab samples (containing bacteria) that were seeded on flat bottomed tissue culture plates for 3-4 hr at 37°C in a humidified incubator were challenged with antibiotic for 12 hr at various concentrations in TSB. Optical density at 595 nm (OD 595 nm) was used to quantify bacterial growth. The community minimal inhibitory concentration (CMIC) is defined as the lowest concentration of antibiotic that resulted in ≤50% increase in OD 595 nm compared to that before introduction of antibiotic.

### 2.11. Bacterial Staining

Frozen sections of chronic wound tissues were stained using ViaGram Red + Bacterial Gram-Stain and Viability Kit (Life Technologies, Carlsbad, CA) with modifications to the manufacturer's protocol. Briefly, the frozen tissue sections were washed in 1X PBS for 5 minutes at room temperature to remove the OCT. Sections were then incubated in wheat germ agglutinin (WGA) conjugate stock solution for 5 minutes. The WGA solution was drained off the slide followed by the addition of 2.5 *µ*L of the DAPI/Sytox Green working solution for 10 minutes at room temperature. The excess working solution was then removed from the section. Sections were mounted and visualized using a Nikon Microphot-FXA microscope with a Nikon DS-Fi1 digital camera.

### 2.12. Scanning Electron Microscopy

Tissues collected were fixed in 4% paraformaldehyde for 4 hrs at room temperature and then processed as described in [19]. Briefly, samples were dehydrated in a series of ethanol for 20 min each followed by critical point drying of the tissues, using Balzers CPD0202 and Au/Pd sputtering in the Sputter coater Cressington 108 auto. The samples were imaged using an XL30 FEG scanning electron microscope.

### 2.13. Biofilm Carbohydrate Composition

Chronic wound swab samples were washed with 80% and 100% ethanol to eliminate low molecular weight components and then with 2 : 1 (v/v) chloroform : methanol to remove lipids and acetone in preparation for drying. Samples were desiccated under vacuum in the presence of P_2_O_5_. Total protein content of the dried swab sample was estimated by the Lowry protein assay. Total carbohydrate content of dried swab sample was estimated by colorimetric phenol-sulfuric acid assay [20], using gum arabic as the standard. For glycosyl composition analysis, dried swab sample was cleaved by trifluoroacetic acid hydrolysis and the resulting monosaccharides were derivatized by methanolysis, N-acetylation, and trimethylsilylation as described [21, 22] with minor modifications. Gas chromatography-flame ionization detection and gas chromatography-mass spectrometry were performed as previously described [23]. DNA was extracted using the DNeasy Blood and Tissue kit (Qiagen, Chatsworth, CA) according to the manufacturer's instructions. DNA concentration was measured and purity confirmed by calculating the OD 260/OD 280 absorption ratio.

### 2.14. Second Harmonic Generation (SHG) Imaging

SHG imaging was done as previously described [10]. Equipped with an NLO interface for a femtosecond Titanium-Sapphire laser excitation source (Chameleon-Ultra, Coherent, Incorporated, Santa Clara, CA) for multiphoton excitation, an inverted Zeiss LSM 510 NLO META laser scanning microscope (Carl Zeiss Microscopy, LLC, Thornwood, NY) for transmitted light and epifluorescence was used. The Chameleon laser provided femtosecond pulses at a repetition rate of about 80 MHz, with the center frequency tunable from 690 to 1040 nm. A long working distance objective (Zeiss, 40X water, N.A. 0.8) was used to acquire images. The sample two-photon signals were epicollected and discriminated by the short pass 650 nm dichroic beam splitter. A META detection module with signals sampled in a 394–405 nm detection range (lex = 800 nm) was used to collect the SHG images. Each image presented in this work is 12 bit, 512 × 512 pixels representing 225 mm × 225 mm field of view.

### 2.15. Statistical Analysis

We used Graphpad Instat Software and Sigmaplot Software. Analysis of variance (ANOVA) was used to test significance of group differences between two or more groups. In experiments with only two groups, we used the Student's *t*-test. Because the differences we observe are not small, we perform experiments in groups of three mice and then repeat the experiment in groups of three as many times as needed to be confident of the results. For the majority of the cases, 2 sets of experiments to a total of 6 animals were sufficient to achieve significant results.

## 3. Results

### 3.1. Impaired Healing and Redox Imbalance Early Post-Wounding in db/db Mouse Wounds

**Figure 1:**
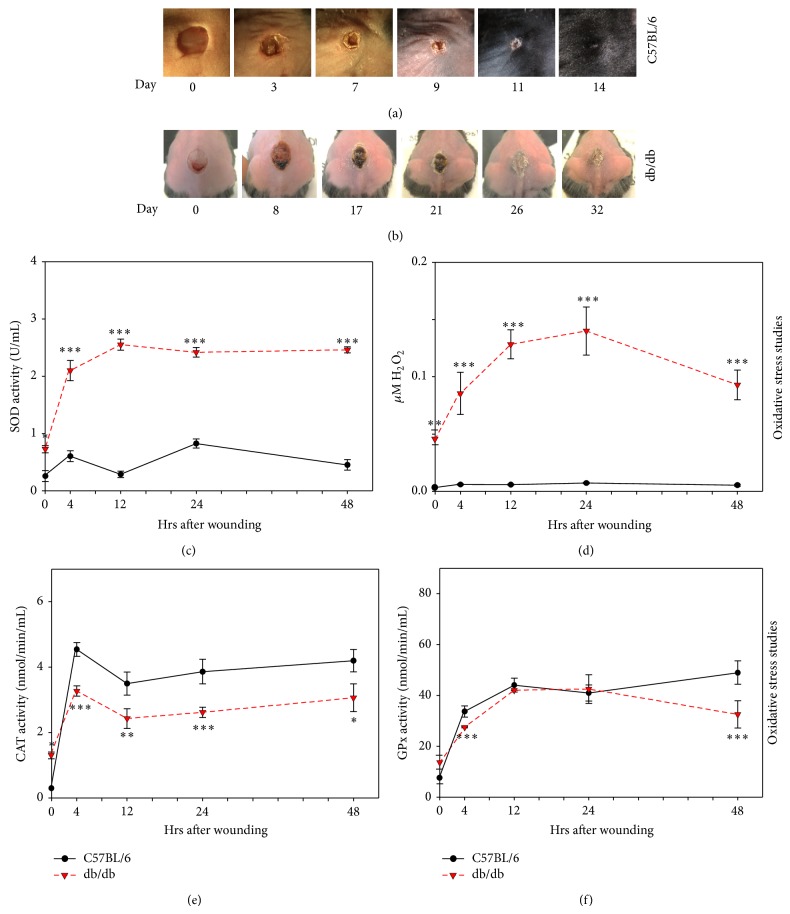
db/db mouse wounds have increased oxidative stress and delayed healing: time course of wound closure in C57BL/6 mice (a) and in db/db mice (b). Wound areas were traced and analyzed using Image J and show delayed closure as compared to C57BL/6. (c) SOD activity was measured using tetrazolium salt that converts into a formazan dye detectable at 450 nm. SOD activity was significantly elevated in the db/db wounds. (d) H_2_O_2_ measurements were based on the peroxidase-catalyzed oxidation by H_2_O_2_ and fluorescent product resorufin read fluorometrically at 530 nm/605 nm. H_2_O_2_ levels were significantly higher in the db/db wounds, confirming the elevated SOD activity in the early hours after wounding. (e) Catalase activity was measured by an enzymatic reaction spectrophotometrically detected with the chromogen purpald at 540 nm and showed reduced activity in the db/db wounds, suggesting a buildup in H_2_O_2_. (f) GPx activity was measured by a coupled reaction with glutathione reductase where GPx activity was rate limiting and absorbance was read at 340 nm per 1 min intervals. GPx activity showed significantly lower levels at 4 hrs and 48 hrs after wounding. These levels confirm improper detoxification of H_2_O_2_ leading to redox stress. Time zero represents unwounded skin. *n* = 6. All data are mean ± SD. ^*^ *P* < 0.05, ^**^ *P* < 0.01, ^***^ *P* < 0.001. *n* = 6 for each of the studies unless indicated differently.

Both db/db and C57BL/6 mice were wounded as described in the Methods section. Wounds were imaged periodically to record wound closure. The C57BL/6 wounds closed in about 11 days whereas the db/db mouse wounds took up to 32 days to close, with flaky and crusty appearance (Figures 1(a) and 1(b)). We examined levels of oxidative stress in these wounds by measuring the detoxifying capacity of SOD. SOD activity was significantly elevated in db/db mice compared to C57BL/6 mice (Figure 1(c)), as was the product of this detoxification process, H_2_O_2_ (Figure 1(d)). However, catalase activity was significantly lower in db/db mice than that of the C57BL/6 (Figure 1(e)) whereas GPx activity was slightly lower very early and more so at 48 hrs (Figure 1(f)). These results show an increase in oxidative stress very early after wounding in the db/db mouse wounds. The nonwounded skin of the db/db mice (*t* = 0) already has exacerbated levels of oxidative stress (Figures 1(c) and 1(d)) which correlates well with the impaired healing these mice exhibit. This led us to hypothesize that high oxidative stress levels in the wound tissue critically contribute to impaired healing and that exacerbated oxidative stress contributes to chronic wound development.

### 3.2. Manipulating the Redox Microenvironment Leads to Chronicity

A chronic wound is one that “has failed to proceed through an orderly and timely reparative process to produce anatomic and functional integrity or that has proceeded through the repair process without establishing a sustained anatomic and functional result” [24, 25]. In humans these wounds stay nonhealing for at least 3 months [24] whereas in animals it has been difficult to establish how long wounds need to be impaired to be considered chronic. However, in general, wounds that do not close by the normative period of time and show minimalistic healing by 26 days have been considered chronic [26]. To test our hypothesis we significantly increased oxidative stress in the db/db wounds by further inhibiting, at the time of wounding, both catalase and GPx activity, two potent antioxidant enzymes. The mice were wounded and treated as described in Methods section under Chronic Wound Model. 3-Amino-1,2,4-triazole (ATZ) was chosen to inhibit catalase because this inhibitor binds specifically and covalently to the active center of the enzyme and inactivates it [27]. Mercaptosuccinic acid (MSA) was chosen to inhibit GPx because the thiol moiety binds to the selenocysteine active site of this enzyme and inactivates it [28]. ATZ has been shown to reduce catalase activity when injected intraperitoneally in rats and mice [29–31]. In rats, the inhibition of catalase activity in liver and kidney was shown to return to normal levels by day 7 after one IP dose of inhibitors [29, 30]. MSA, also known as thiomalate, is the most potent of all mercaptans for inhibiting GPx. It has been shown to clear from the plasma in a matter of a few hours to a couple of days [28]. The specific doses described in the Methods section were chosen after an extensive literature search to determine what has worked in mice for effective inhibition of these enzymes without causing major side effects [32–35]. Moreover, the treatment with inhibitors affects the breakdown of H_2_O_2_, a product of the oxidative burst generated by neutrophils that are present early after wounding. As a consequence, inhibition of the two antioxidant enzymes increases the strength of oxidative burst without affecting neutrophil activity.

**Figure 2:**
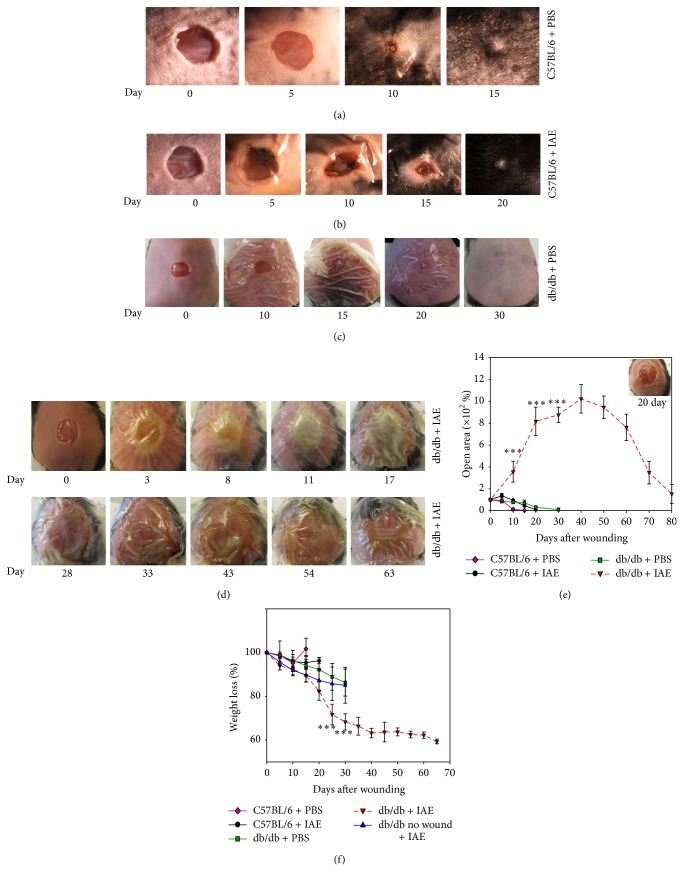
Redox imbalance leads to chronic wound development. (a) Wound closure in C57BL/6 mice treated with PBS occurs by 15 days after wounding. (b) C57BL/6 mice treated with inhibitors heal by 20 days after wounding. (c) Db/db mice treated with PBS heal completely by 30 days. (d) Db/db mice treated with inhibitors become chronic and do not heal for as long as 100 days. (e) Percent open wound area over time in wounds of C57BL/6 and db/db mice after treatment with PBS or IAE. (f) Weight loss in C57BL/6 and db/db mice after PBS and IAE treatment. *n* = 7 for each treatment except for db/db mice treated with IAE; we have now treated well over 100 mice. All data are mean ± SD. ^*^ *P* < 0.05, ^**^ *P* < 0.01, ^***^ *P* < 0.001.

Control C57BL/6 mice treated with either PBS or the inhibitors for the two antioxidant enzymes (IAE) closed by day 12 and 20, respectively (Figures 2(a) and 2(b)). The wounds in the IAE treated mice took longer to close and initially appeared to be ulcerous but did not form exudate. db/db mice treated with PBS healed by 30 days and did not form exudate or become ulcerous (Figure 2(c)). The wounds of the db/db treated with IAE, however, became ulcerous, developed exudate, and remained open as long as 100 days and sometimes more (Figures 2(d) and 2(e)). The areas of the wounds were measured by removing the tegaderm, lightly cleaning the wound, and taking a picture before covering the wound again with tegaderm (inset in Figure 2(e) shows a picture of such wounds). As is observed in humans with chronic wounds [36], the weight of db/db mice with chronic wounds steadily decreased by as much as 40% by day 40 after wounding (Figure 2(f)). This was not primarily due to treatment with the inhibitors because db/db mice treated with the IAE without wounding lost only weight until day 15 and then began to recover whereas the mice with chronic wounds continue to dramatically lose weight (Figure 2(f)).

### 3.3. Chronic Wounds in db/db Mice Sustain Complex Microbiota in the Presence of Redox Imbalance

**Figure 3:**
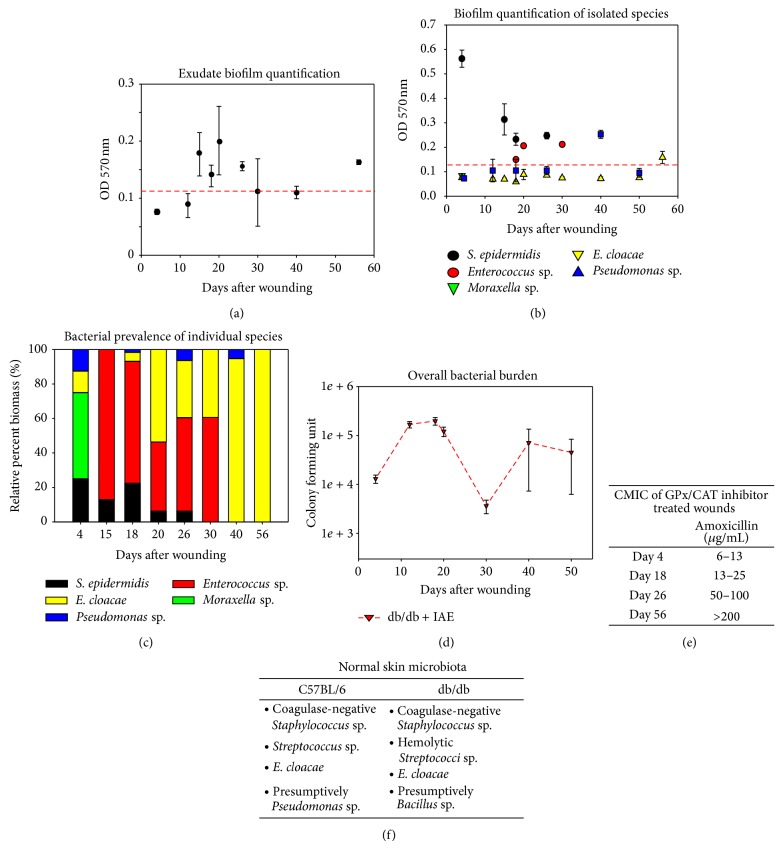
Chronic wounds contain complex antimicrobial-resistant wound microbiota. (a) Bacterial contents of in the swab sample were quantified by measuring the optical densities of stained bacterial films at OD 570 nm (≥0.125 is considered to be biofilm positive—dashed red line). (b) Specific bacterial strain identification shows a dynamic presence of different species with biofilm-forming-capacity. (c) Bacterial prevalence of individual species shows the changing dynamics of the wound microbiota. Quantifying the relative percentage of individual bacterial species demonstrated that* Enterococcus* sp. and* S. epidermidis* made up the large majority of the microbial mass with traces of* Pseudomonas* and* E. cloacae* at 4 days after IAE treatment. At day 20, the majority of the biomass was composed of biofilm-producing* Enterococcus* sp. (~40%) and non-biofilm-producing* E. cloacae* (50%). By day 30, biofilm-producing* S. epidermidis* disappeared. At day 40, the wounds progressively advanced toward a monospecies infection dominated by* E. cloacae* and to lesser extent by biofilm-producing* Pseudomonas*. By day 56, the wounds are exclusively colonized by biofilm-producing* E. cloacae*. (d) Bacterial burden was evaluated by colony forming unit counts in db/db wounds treated with IAE. (e) The community minimum inhibitory concentration (CMIC) on wound the wound swab samples was examined using the antibiotic amoxicillin. With time, the resistance increased. (f) Skin swabs were collected from C57BL/6 and db/db mice to evaluate their normal skin microbiota. Very similar bacteria were found in the skin of both mouse strains. All data are mean ± SD. *n* = 6 for each of the studies unless indicated differently.

Microbial infection of wounds in db/db mice was first observed about 4 days after treatment with IAE and persisted for at least 56 days in most animals (Figure 3(a)); the presence of biofilm-associated microbial infection was first seen about 15 days after wounding. Bacterial isolation and characterization showed that the samples collected from the wounds are comprised of many bacterial species and contains both biofilm- and non-biofilm-producing species (Figure 3(b)). Interestingly, monospecies cultures revealed that* S. epidermidis* isolated from day 4 samples produces biofilm when grown independently from the community, suggesting that communal interactions may be an important regulator of biofilm production. As the infection progresses, the bacterial composition evolves from several species of nonbiofilm producers (day 4) to progressively fewer species at day 15 that are biofilm producers. By day 30 only 2 species remained (*Enterococcus* sp. and *E. cloacae*) and by day 56 the only bacterium remaining was* E. cloacae* which by that time had become biofilm producing (Figures 3(b) and 3(c)). Furthermore, evaluation of chronic wound tissues to obtain the bacterial profile in the wound bed showed that the profile was closely similar to the bacterial species in the wound bed collected in the swab sample, with only minor differences (e.g., relative abundance among bacterial species).

Impaired healing has been shown to be associated with high bacterial burden [37]. Counts of Colony Forming Units (CFU) from wound swab samples revealed that bacterial burden was at its highest at 20 days after wounding (1.4 ± 0.1 × 10^5^ CFU), thereby pushing the wound into a state of chronicity. At day 30, there was a reproducible dramatic decrease in bacterial burden (approximately 28X) compared to day 20 that coincided with the disappearance of* S. epidermidis* and of* Pseudomonas* sp. However, by day 40, wound bioburden was dominated by* E. cloacae* and bioburden had recovered to a level comparable to day 20 and remained relatively unchanged thereafter (Figure 3(d)).

### 3.4. Chronic Wound Microbiota Is Resistant to Antibiotic Challenge

Microbial communities living within biofilms are often resistant to antibiotics [38]. Quantifying the microbial community minimal inhibitory concentration (CMIC) of amoxicillin required to inhibit the growth of the microbial community demonstrated that biofilm positive bacterial communities appearing at 15 days and after are more resistant to antibiotic killing, and this resistance increased with time (Figure 3(e)). Biofilm negative bacterial communities collected at 4 days after wounding showed CMIC of less than 15 *μ*g/mL and, by day 56, these communities were more than 200 *μ*g/mL and the biofilm-forming bacteria.

To determine whether normal skin microbiota can be the source for infection, we took skin swabs from unwounded C57BL/6 and db/db mice and cultured the skin microbial flora (Figure 3(f)). The majority of the bacteria found on both C57BL/6 and db/db skins belonged to the Firmicutes phylum, specifically* Staphylococcus* sp. and* Streptococcus* sp. We were also able to isolate bacteria from the Proteobacteria phylum (specifically* Bacillus Pseudomonas* and* Enterobacter*) (Figure 3(f)). The bacterial species that colonized C57BL/6 and db/db wounds were similar but the db/db skin on average has higher bacterial density than C57BL/6 (not shown). Furthermore, these bacteria are known to be similar to normal human skin microbiota [39]. Thus, skin microbiota of nonwounded skin in our model is very similar to that of humans and is a contributor to the biofilm found in chronic wounds. It is notable that the bacterial profile isolated from the skin of db/db mice was similar to that observed using near-full-length 16S rRNA sequencing for bacterial species that colonized db/+ and db/db unwounded skin [40].

### 3.5. Treatment with Antioxidant Agents Reverses Chronicity

In this model system, the wounds are chronic by 20 days after wounding and after treatment with IAE. To reverse chronicity, we treated the animals with the antioxidant agents (AOA) NAC and *α*-toc. NAC is produced by most cells in the body; hence we applied it locally to the wound every day. This approach has been shown to be effective in diabetic mice [41]. *α*-toc, on the other hand, is produced primarily by the liver and circulates throughout the body. Therefore, we applied it IP [42, 43], simulating systemic delivery in humans. The doses we used are described in Materials and Methods. The treatment doses for *α*-toc and NAC were chosen after extensive literature searches [44–46].

**Figure 4:**
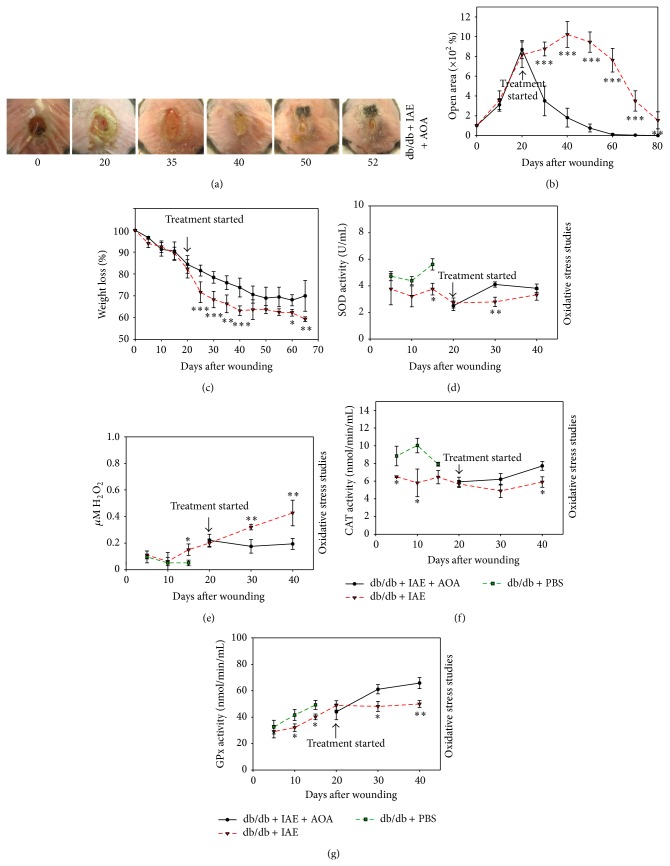
Treating chronic wounds with AOA leads to proper healing and reduced oxidative stress. (a) Chronic wounds of db/db mice treated with IAE and then AOA, NAC, and *α*-toc, show a faster rate of wound closure. Wound areas were traced and analyzed using Image J. (b) Percent open wound area in db/db wounds treated with IAE and then AOA compared to db/db wounds treated with IAE alone. AOA significantly accelerated wound closure. (c) Weight loss was significantly higher in db/db mice treated with IAE alone than when treated with IAE and then AOA. (d) The levels of SOD in db/db wounds treated with PBS were significantly higher than db/db wounds treated with IAE suggesting that the latter have reduced dismutation of O_2_^∙−^ radicals and accumulation of reactive radicals. AOA treatment of chronic wounds significantly increased SOD activity 10 days after treatment suggesting increased dismutation of O_2_^∙−^ radicals. (e) Increases in H_2_O_2_ levels (examined as described in Figure 1) in db/db wounds treated with AOA were stabilized in comparison to the increasing stress in the non-AOA treated wound. (f) Catalase activity was significantly increased in PBS treated wounds in the first 15 days as compared to the IAE treated wounds. AOA treatment of chronic db/db wounds significantly increased catalase activity by 20 days of treatment. (g) GPx in the db/db mice treated with PBS was significantly higher than the db/db mice treated with IAE. Enzyme activity in the db/db chronic wounds treated with AOA was significantly higher than db/db mice with only IAE. The overall effect was stabilized levels in H_2_O_2_. All data are mean ± SD. ^*^ *P* < 0.05, ^**^ *P* < 0.01. *n* = 6 or 7 for each of the studies unless indicated differently.

We found that healing improved dramatically by 30 days after AOA treatment (50 days after wounding) as compared to the wounds treated with vehicle that can take up to 100 days to close (compare Figures 4(a) and 4(b) with Figures 2(a)–2(e)). Moreover, the weights of the db/db mice treated with AOA began to stabilize starting at 20 days after the initiation of treatment (40 days after wounding) (Figure 4(c)). These results, coupled with our finding that when the mice are treated only with inhibitors without wounding they do not lose weight past day 15 or so, indicate that the great loss of weight is due to the wounds and not to the treatment with IAE (compare Figure 4(c) with Figure 2(f)). The AOA were also able to restore the detoxifying capability of catalase and GPx to reduce oxidative stress. SOD increased within 10 days after treatment with AOA (30 days after wounding) (Figure 4(d)). Also, H_2_O_2_ levels decreased by day 10 after treatment compared to increasing H_2_O_2_ levels in chronic wounds (Figure 4(e)) and both catalase and GPx enzyme activity increased following treatment whereas they were approximately constant in chronic wounds (Figures 4(f) and 4(g)).

**Figure 5:**
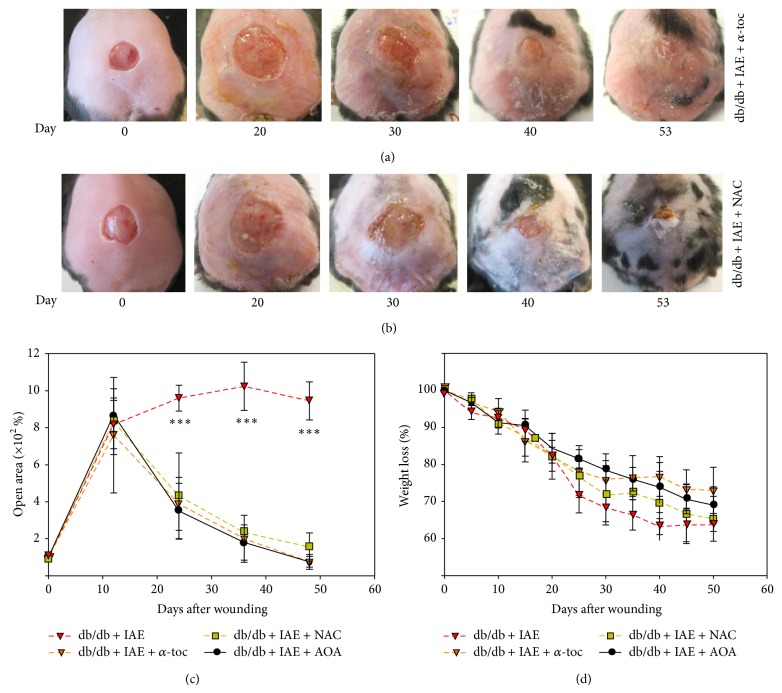
Chronic wounds treated with individual antioxidants. (a) Wound closure in db/db chronic wounds treated with AOA *α*-toc was observed by day 53 after wounding. (b) Wound closure in db/db chronic wounds treated with AOA NAC was observed by day 53 after wounding. (c) Percent open wound area was calculated using Image J. Open area was significantly decreased in db/db wounds treated with IAE and then AOA. (d) Mouse weight loss was significantly higher in animals with no AOA treatment. *n* = 5. All data are mean ± SD. ^*^ *P* < 0.05, ^**^ *P* < 0.01, ^***^ *P* < 0.001.

**Figure 6:**
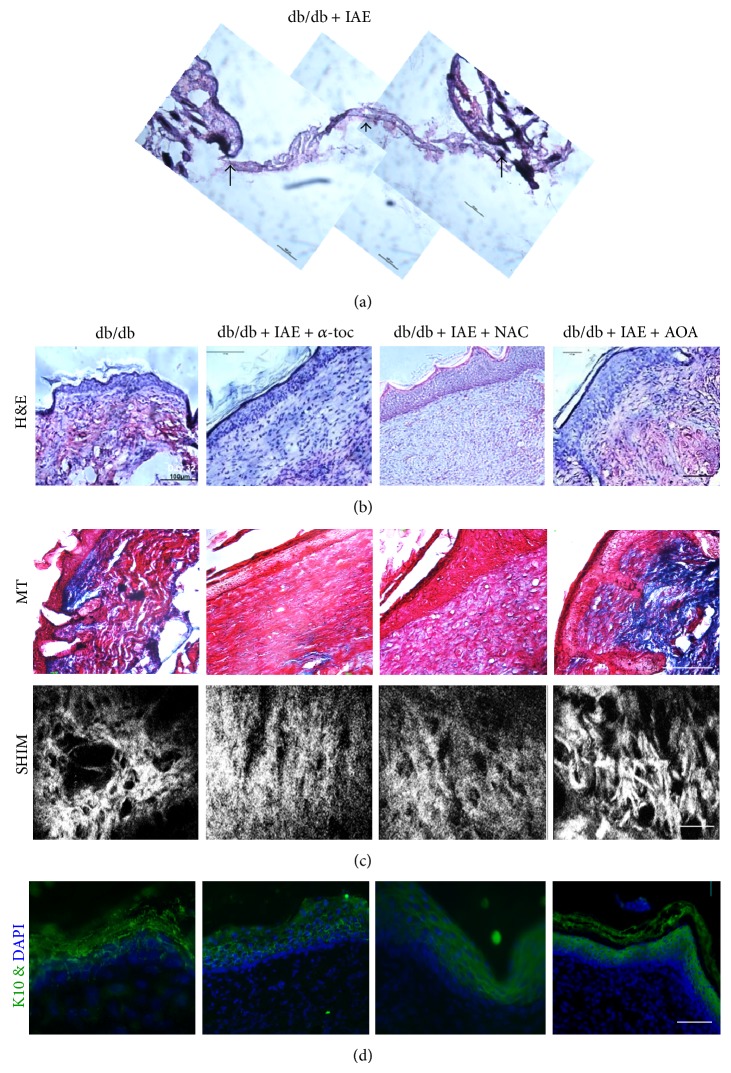
Histological evaluation of normal, chronic, and treated wounds: (a) H&E-stained section of day 64 chronic wound in db/db mice treated with IAE. (b) H&E-stained sections of representative animals to illustrate the histology at wound closure of db/db wounds from a nontreated animal (panel 1), an animal treated with IAE, and then with AOA *α*-toc (panel 2), an animal treated with IAE, and then with AOA NAC (panel 3) and an animal treated with IAE and then both AOA (panel 4). Scale bar 100 *µ*m. (c) Representative Masson-trichrome staining (blue color) illustrating loss of collagen deposition in nontreated db/db mice (panel 1). A decrease in collagen deposition was seen in chronic wounds treated with AOA individually (panels 2 and 3). Chronic wounds treated with both AOA had a significant increase in collagen deposition and fibril formation (panel 4). Second Harmonic Generated Imaging (SHIM) shows abnormalities in collagen bundles in the nontreated db/db wounds whereas no clear fibers are seen in individual AOA treated wounds. In wounds with both AOA, fibers are well formed and appear more mature. For SHIM the scale bar is 10 *µ*m. (d) Keratin 10 illustrates the presence of the epithelial cytoskeleton in the terminally differentiating epithelial cells found in the suprabasal layer. The epithelium is much more mature in the wounds treated with both AOA. Scale bar is 100 *µ*m.

To test whether the two AOA are needed together to reverse chronicity, we treated the chronic wounds individually with NAC (Figure 5(a)) or *α*-toc (Figure 5(b)). Visual examination of the wounds treated with single antioxidants shows little difference from those treated with both together (compare Figures 5(a) and 5(b) with Figure 4(a)). Quantitatively, *α*-toc was a close approximation to the two agents together for wound closure; NAC was slightly less effective (Figure 5(c)). Similarly, for weight loss, *α*-toc closely matched combined agents and NAC was slightly less efficient (Figure 5(d)). These findings suggest that either of the AOA is able to reverse the chronic state. However, these measurements of macroscopic parameters are insufficient to adequately characterize reversal of chronicity. As shown in Figure 6, the structure of the wound tissue is immature when the mice are treated with the individual AOA but not when treated with both.

Histological examination of db/db chronic wounds shows a complete loss of tissue structure; there is no epidermis and all that is present of the granulation tissue is a very thin band of undifferentiated tissue (Figure 6(a); shown between the arrows). Therefore, this tissue cannot be analyzed further. Db/db nonchronic wounds after closure showed that the granulation tissue is poorly developed (Figure 6(b), first panel). Chronic wounds treated with NAC or *α*-toc individually (Figure 6(b), panels 2 and 3) are much improved but their epidermis and granulation tissue are immature. In contrast, chronic wounds treated with both antioxidants (Figure 6(b), panel 4) show a well-developed granulation tissue and thinner epidermis containing rets. Moreover, Masson trichrome (MT) stain and Second Harmonic Imaging Microscopy (SHIM) for collagen deposition and structure revealed a similar pattern (Figure 6(c)); the overall deposition of fibrillar collagen in the wounds treated with both AOA was better than those of db/db nonchronic wounds and chronic wounds treated with either of AOA alone (Figure 6(c) compares panel 4 with 1–3). As shown by keratin 10 staining (Figure 6(d)), the epidermis was well organized and thinner in the animals treated with both AOA (panel 4) than in the nonchronic db/db and those treated with the AOA individually (panels 1–3).

### 3.6. Controlling Redox Stress Leads to Decrease in Biofilm Production and Increased Sensitivity to Antibiotic

**Figure 7:**
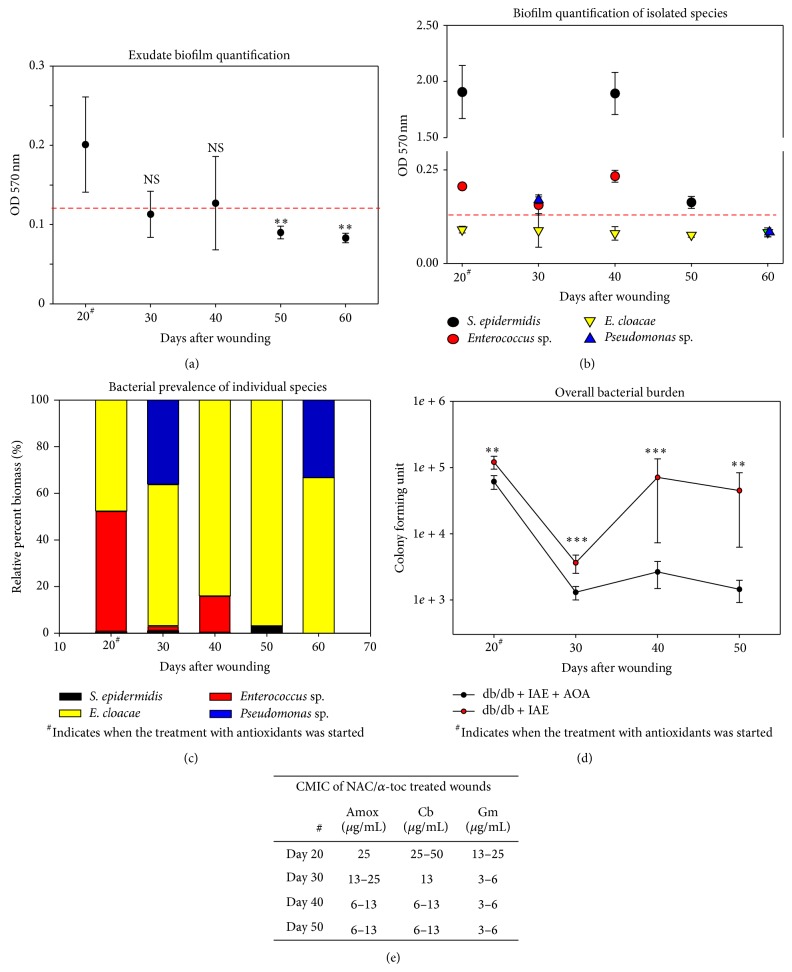
AOA treatment reduces biofilm-forming microbiota and increases bacterial antibiotic susceptibility: (a) The bacterial contents of the swab samples were evaluated by measuring optical densities at OD 570 nm. AOA treatment reduced biofilm formation. (b) Individual bacterial colonies with biofilm-forming-capacity were quantified by measuring the optical densities and were also reduced with AOA treatment. (c) Bacterial prevalence of individual species was again reduced with AOA treatment. (d) Bacterial burden measured by colony forming unit counts was significantly reduced in AOA treated db/db wounds. (e) Community minimum inhibitory concentration (CMIC) of bacteria present in wounds treated with AOA was performed using gentamicin (Gm), carbenicillin (Cb), and amoxicillin (Amox). CMIC degreased significantly with time of AOA treatment. *n* = 5. All data are mean ± SD. ^**^ *P* < 0.01, ^***^ *P* < 0.001. *NS* = nonsignificant.

After 10 days of AOA treatment (day 30 after wounding), biofilm-producing capacity of the chronic wound microbial flora is reduced by approximately 30% (Figure 7(a)). By day 50 after wounding the microbial flora was considered to be negative for biofilm production (OD 570 nm < 0.125). Similarly, microbial profiling showed that by day 50 the overall biofilm-producing capacity of the individual bacterial species was reduced markedly (Figure 7(b)). This is most noticeable by the drastic decrease in biofilm production by* S. epidermidis* at day 50 compared to day 20 and the loss of biofilm production by* Pseudomonas* sp. at day 60 compared to day 30. The use of AOA treatment affected the bacterial composition of the wounds, most notably by the reduction and then elimination of* Enterococcus* sp., the increasing prevalence of nonbiofilm producing* E. cloacae*, and the colonization by* Pseudomonas* sp. (Figure 7(c)). CFU counts (Figure 7(d)) revealed a marked decrease in bacterial burden at 10 days of AOA treatment that, in contrast to non-AOA-treated wounds, remained relatively unchanged thereafter.

To determine whether the reduction in biofilm production resulting from AOA treatment enhances the antimicrobial effect of amoxicillin, CMICs of wounds treated with NAC and *α*-toc were evaluated (Figure 7(e)). After 10 days of antioxidant treatment, the CMIC for amoxicillin was reduced significantly. Similar reduction was observed with other antibiotics, such as carbenicillin and gentamicin (Figure 7(e)). These observations confirm that abating redox stress with NAC + *α*-toc alters the wound environment resulting in a bacterial phenotype that produces less biofilm and is more sensitive to antibiotics.

**Figure 8:**
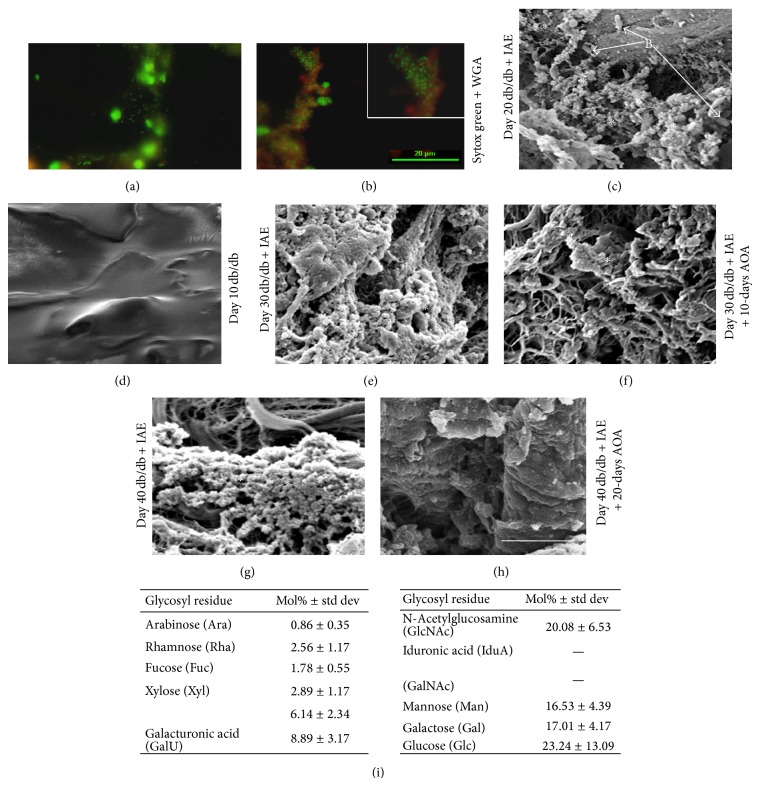
Morphological characterization of biofilm in chronic and AOA treated wounds. (a, b) Fluorescent microscopy of frozen sections of chronic wounds IAE treated at 20 days after wounding, stained by Sytox green for bacterial DNA and wheat germ agglutinin conjugated with Texas Red for the tissue and biofilm matrix, shows presence of biofilm. (c) SEM shows bacteria embedded in a biofilm-associated matrix (stars) with presence of bacteria [B] in chronic wounds at day 20 after wounding and treatment with IAE. (d) Day 10 db/db wounds without inhibitors to illustrate how the tissue looks like in non-IAE treated wounds. (e) Chronic wounds treated with IAE at day 30 after wounding show more biofilm than at day 20 (c) and much more than the day 30 wounds that were treated with AOA for 10 days (f). (g) Chronic wounds treated with IAE at day 40 after wounding show more biofilm than at day 30 (e) and much more than the day 40 wounds that were treated with AOA for 20 days (h). Scale bar for (c–h) is 5 *µ*m. (i) Biochemical analysis of the biofilm obtained from chronic wounds.

To further confirm the presence of biofilm-forming bacteria in chronic wounds and the decrease in colonization upon AOA treatment, we performed both light microscopy with fluorescent stainings and scanning electron microscopy (SEM). For the light microscopy we stained frozen sections of chronic wound tissue (IAE treated) at 20 days after wounding with a combination of Sytox green, which has high affinity for nucleic acids, and wheat germ agglutinin (WGA) conjugated with Texas Red which labels ECM molecules including biofilm. Both Gram-negative and Gram-positive bacteria in the tissue appear as very small green specks because their nuclei are stained with the Sytox green. They are associated and embedded in the red matrix stained by WGA (Figures 8(a) and 8(b)). As previously reported, although Sytox Green and WGA stain both host and bacteria, the size and morphology of the cells enabled distinguishing the presence of bacteria from host cells [47]. This technique is now considered as one of the staining techniques for biofilms in tissue specimens [48]. The SEM pictures of chronic wounds (IAE treated) show an abundance of bacteria at 20 days after wounding when compared to a normal db/db wound at 10 days (Figures 8(c) and 8(d)). We used 10 days for the db/db nonchronic wounds as control because biofilm is already seen by day 10 in wounds treated with IAE. Bacteria were embedded in a biofilm-like matrix (stars) covering the wound (Figure 8(c)). Upon treatment with AOA for 10 days (30 days after wounding), there was a reduction in the biofilm matrix seen in the wound (compare Figure 8(e) with Figure 8(f)). Furthermore, by 20 days of AOA treatment, there was further decrease in the presence of matrix and bacteria when compared to the increase in biofilm formation observed in the chronic wound (compare Figure 8(g) with Figure 8(h)). Following bacterial species identification and confirmation of presence of biofilms in the chronic wound, we evaluated the composition of the biofilm (Figure 8(i)). Carbohydrate analysis using gas chromatography shows the various carbohydrates forming the biofilm. Total protein content of the dried biofilm in swab sample was 44.6% ± 15.1% (w/w) and total DNA content was 1.55% ± 0.19074% (w/w). Total carbohydrate content of the dried biofilm in the swab sample was analyzed by colorimetric phenol-sulfuric acid assay was 1.98% ± 0.45% (w/w) and by gas chromatography analyzing glycosyl composition was 2.74% ± 1.46% (w/w). The latter showed that N-acetylglucosaminyl (GlcNAc), galactosyl, mannosyl, galacturonosyl, and glucuronosyl were the major glycosyl residues in the dried biofilm. Neither iduronosyl nor N-acetylgalactosaminyl residues were detected in the biofilm. Among the glycosyl residues, the relatively high amounts of N-acetylglucosaminyl and mannosyl residues were consistent with the presence of N-glycan type glycoproteins. Other glycosyl residues, particularly galacturonosyl residues, seemed to be of other origins.

## 4. Discussion

**Figure 9:**
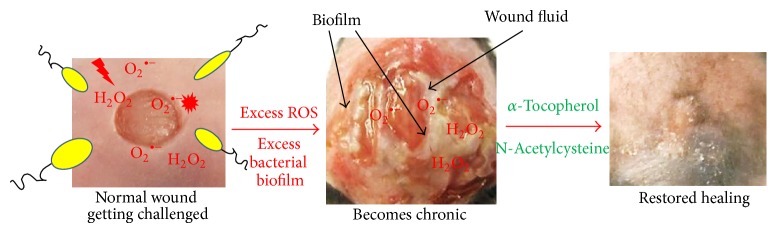
Schematic illustration of our chronic wound development model and how chronicity is reversed. A wound is created in the presence of excessive levels of reactive oxygen species by inhibiting key antioxidant enzyme (IAE) activity. These wounds become chronic and produce wound exudate that contains bacterial biofilms. Reversal of chronicity towards normal healing was achieved by application of antioxidant agents (AOA) *α*-toc and N-acetyl cysteine (NAC).

We show here that we can generate chronic wounds in a diabetic mouse model of impaired healing by creating high levels of oxidative stress in the wound tissue using IAE at wounding. The wounds remain open for 80–100 days or longer. We also show that chronicity of these wounds can be reversed by application of appropriate AOA, leading to healing. These findings are summarized schematically in Figure 9. Briefly, we find that H_2_O_2_ is elevated very early after wounding of these mice and that the antioxidant enzyme activity is significantly lower than in wounds of normal mice, suggesting an increase in oxidative stress in the wound tissue. Accordingly, we developed a protocol in which the oxidative stress environment in these impaired wounds is further increased by inhibition of the two critical antioxidant enzymes, catalase and GPx. These wounds go on to form chronic ulcers within 20 days of this treatment. The ulcers remain open for months and develop a complex microbiota of biofilm-forming bacteria that are also found in chronic wounds in humans. Chronicity was reversed by treatment of the chronic wounds with the antioxidant agents NAC and *α*-toc. Under these conditions, the oxidative stress falls rapidly, the biofilm dismantles, and the wound heals. This, to the best of our knowledge, is the first chronic wound model in which biofilm develops spontaneously allowing for longitudinal assessment of the wound microbiome in its natural environment. These findings define a unique relationship between redox imbalance and complex biofilm development in wound chronicity, providing an opportunity to understand the mechanisms of action of both and potentially for the development of new therapies for humans.

There are many similarities between the development of chronicity in these mice and similar chronic wounds in humans, especially nonpressure diabetic ulcers. For example, mice with chronic wounds lose considerable weight, as also occurs in many humans suffering from chronic wounds [49]. Also, a very complex microbiota spontaneously develops in these mice wounds, as it does in humans wounds [50].* Staphylococcus epidermidis* and other coagulase-negative staphylococci exist as harmless or even beneficial commensal bacteria of the skin. However, in diabetic, elderly, and immobile individuals, these commensal bacteria can cause diseases and are responsible for a large percentage of infection in wounds that do not heal [51]. In our animal model, microbial flora that colonizes the db/db mouse wounds is identified as* S. epidermidis*,* Enterococcus* sp.,* Enterobacter cloacae*, and* Pseudomonas* sp., and they exist as a complex and dynamic community. Although commensal gram-negative bacteria* E. cloacae* are not reported from normal human skin, their presence in human wounds comes from gastrointestinal contamination of the skin [51, 52]. Others have shown that microbial flora cultured from patients with venous leg ulcers with or without clinical symptoms is polymicrobic [16, 53]. Furthermore, similar microbiota is found in combat wounds, chronic necrotizing skin diseases, and other chronic wounds [54–56].

The establishment of selective microbial populations toward chronicity is probably due to both bacterial competition and cooperation in concert with the unique clinical and immunological phenotype and pathophysiology of the host wound tissue. In the diabetic mice of our study, chronic wounds evolve into essentially a monospecific infection dominated by biofilm-producing* E. cloacae*. In other species (including humans), the specific biofilm producers may differ but the overall result is likely to be the same. From our observations, the bacterial ecology in our mouse model is polymicrobic and dynamic. The molecular factors and parameters that resulted in the dynamics observed in our mouse model are yet to be defined and require further investigation. Our hypothesis is that due to competition and fitness, certain species will outcompete to predominate in the wounds and establish chronic infection. Therefore, there are differences in the bacterial species that colonize the wound at days 20 and 30. For reasons that we cannot yet explain, the microbial ecology tends to change dramatically around day 30, transitioning from a polymicrobic infection to a monospecific infection.

It is clear from our histological sections that the granulation tissue of the chronic wounds is virtually nonexistent, making it essentially impossible to perform studies to identify inflammatory cells and/or blood vessels. In addition, we observed that the epidermis does not progress to close the wound and, in fact, the wound size increases. This is in agreement with our previously published findings in which cornea excision wounds in mice exposed to second-hand cigarette smoke increased in size [57]. In that case, the smoke exposure created increased stress in the wound that led to inhibition of cornea epithelial migration to cover the wound. Furthermore, the leading edge to the epithelium did not adhere to the underlying cornea stroma. We speculate that similar processes are occurring in the chronic wounds and we are currently investigating these possibilities.

ROS is known to play a critical role in wound healing [15, 58]. Under normal physiological conditions, generation of H_2_O_2_ is seen very early after injury. This occurs in the presence of nicotinamide-adenine-dinucleotide (NADH) dependent oxidases (NOXs) produced by resident endothelial cells and fibroblasts, followed by neutrophils and macrophages [59]. For proper healing, a delicate balance needs to exist between their good and deleterious effects in the wound tissue. A balanced ROS response will clean and inhibit infection while simultaneously triggering signaling mechanisms that stimulate healthy healing [15, 60, 61]. However, elevated levels of ROS can cause a wide variety of tissue damage and lead to impaired healing [62]. Therefore, for a positive role in healing, ROS needs to be in balance to be an effective antimicrobial and signaling agent but yet not to cause destructive effects to the tissue. If not, the high levels of ROS or the reduced levels of antioxidant scavenger molecules such as the antioxidant enzymes catalase and GPx and antioxidant vitamins such as VitE, C and D, will lead to chronic wounds [63]. The data presented here show that exacerbation of the levels of ROS immediately after wounding by inhibiting catalase and GPx causes the wound to become populated with a polybacterial infection that then leads to selection for bacteria that form biofilm, causing wounds with impaired healing to become chronic. The question now is: how does the increase in ROS levels lead to development of biofilm that puts the wound in a state that leads to chronicity? To that end, we currently are investigating the differences in gene expression levels in the presence or absence of IAE in both C57BL/6 and db/db mice (unpublished data). The observed pattern of gene expression, coupled with the fact that some of them are related to changes in oxidative stress, inflammation, apoptosis, and mitochondrial functions that regulate oxidative stress, suggests that these processes are critical for development of biofilm-induced chronicity. We speculate that a variety of genes that are altered when ROS is significantly increased favor the ability of the bacteria to produce virulence factors that allow them to form biofilm.

## 5. Conclusion

We have been successful in creating the first chronic wound model in which biofilm forms naturally without the need of introducing bacteria grown* ex vivo*. In this model, excessive increase in ROS at the time of wounding provides the skin microbiota with favorable conditions to grow and be able to form biofilms (Figure 9). The restoration of the redox balance by application of AOA causes biofilm to disappear from the wounds, resulting in significant improvement in wound healing. Others have proposed that the pathogenesis of chronic wounds is dependent on factors such as tissue hypoxia, bacterial colonization, ischemia-reperfusion injury, and an altered cellular and systemic stress response. We propose that the critical parameter for development of chronic wounds in diabetic humans, and perhaps others, is an excessively high level of reactive oxygen species in the local microenvironment of the wound very early after injury. In this microenvironment, normal skin bacteria that otherwise would not form biofilm colonize the wound and become biofilm producers.
